# Low eligibility for hepatitis B treatment in the Brazilian public health system

**DOI:** 10.1590/0037-8682-0297-2021

**Published:** 2022-04-08

**Authors:** Cristiane Faria Oliveira Scarponi, Marco Antônio Ferreira Pedrosa, Marcos Paulo Gomes Mol, Michael John Mascarenhas Hardman, Dirceu Bartolomeu Greco

**Affiliations:** 1 Fundação Ezequiel Dias, Divisão de Epidemiologia e Controle de Doenças, Belo Horizonte, MG, Brasil.; 2 GeOpem Engenharia e Tecnologia Limitada, Brejo Santo, CE, Brasil.; 3 Fundação Ezequiel Dias, Divisão de Ciência e Inovação, Belo Horizonte, MG, Brasil.; 4 Frimley Health NHS Foundation Trust, Frimley Park Hospital, Camberley, Surrey, England.; 5 Universidade Federal de Minas Gerais, Programa de Pós-Graduação em Ciência da Saúde: Infectologia e Medicina Tropical, Belo Horizonte, MG, Brasil.

**Keywords:** Hepatitis B, Treatment eligibility, Guideline adherence, Antiviral therapy, Public health, Brazil

## Abstract

**Background:**

Chronic hepatitis B (CHB) affects 257 million people worldwide. However, the proportion of patients eligible for treatment in the public health system has not been established. This study describes the clinical and laboratory profiles of untreated CHB patients and estimates the eligibility rate for antiviral therapy in accordance with the Brazilian Clinical Protocol and Therapeutic Guidelines.

**Methods:**

Records of 670 CHB patients were collected from May 2012 to September 2013 in Minas Gerais. Data from each patient were analyzed by hepatitis B virus (HBV) management.

**Results:**

461 CHB patients were treatment-naive. Of these, 23 were HBeAg-positive, 352 were HBeAg-negative, and 14 were clinically diagnosed with cirrhosis. Periodic monitoring was performed in only three patients. However, 9.3% of untreated patients met the eligibility criteria for HBV treatment.

**Conclusions:**

Few CHB patients were active carriers and eligible candidates for antiviral therapy. This study revealed inadequate pre-treatment conduct in the Brazilian public health system, emphasizing the need for regular laboratory follow-up for patients initially not eligible for treatment. Such information may indirectly subsidize the planning and improvement of actions and services related to optimal HBV management in the public sphere.

## INTRODUCTION

Chronic hepatitis B (CHB) affects 257 million people and remains a progressive silent disease. It has a high impact on public health, as most individuals have been asymptomatic for decades, but also has a high capacity to transmit the hepatitis B virus (HBV), thus perpetuating the pandemic. CHB is laboratory-proven by the persistent presence of HBV surface antigen (HBsAg) in serum for more than 6 months, with or without high levels of aminotransferases and histological lesions^1^. According to the Ministry of Health, 120,343 confirmed cases of hepatitis B infection were reported in Brazil between 1999 and 2011. Most of these cases (36.3%) were concentrated in the Southeast Region, with the Minas Gerais state being responsible for 14.4% of the cases. Clinical information was included in 87.8% of reported cases. The chronic form was predominant (78.3%), which highlights its impact in Brazilian territory^2^.

Currently, there are several published consensus guidelines to assist physicians in the most appropriate CHB management by providing recommendations for screening, diagnosis, monitoring, and treatment indications. In general, they focus on simple or more elaborate diagnostic tests, both invasive and non-invasive, in order to determine the current stage of liver disease so that patients can be promptly evaluated for HBV treatment. Antiviral treatment eligibility was directed according to the guidelines’ pre-established criteria, prioritizing mainly patients with more advanced liver disease and a higher risk of mortality^3^ ^-^ ^7^. However, patients who do not meet the treatment criteria should be monitored as they may change their disease state throughout their life and become eligible for antiviral therapy at another time. Although CHB management guidelines are frequently cited, it is not clear how these recommendations are applied in daily clinical practice, regardless of the infrastructure provided by the local health system and available resources^8^.

In 2002, the Brazilian Health Ministry created the “Clinical Protocol and Therapeutic Guidelines for the treatment of Chronic Viral Hepatitis B and Co-infections (CPTG-CHB),” which aimed to cover professionals at all care levels due to the variable access to health services and the significant impact of hepatitis B across the country^9^. Subsequent updates were based on scientific evidence and international consensus and structured through basic algorithms to facilitate the rational use of the therapeutic arsenal and laboratory tests provided by the Unified Health System (SUS). However, the true adherence rate to the CPTG-CHB recommendations in clinical practice is not known, nor is the number of patients eligible for antiviral therapy. Furthermore, studies describing the profile of people living with HBV who seek care in the Brazilian public health system are rare^10^.

Universal and comprehensive care may provide opportunities to save lives, improve the patient’s clinical condition, and reduce the incidence and transmission of HBV. However, it also presents practical challenges for public policymakers and implementers in low-and middle-income countries. The low coverage of laboratory tests, treated patients, and care services are important gaps to be addressed in order to achieve global HBV elimination targets by 2030^1^. Therefore, this study aimed to describe the predominant laboratory and clinical profiles of untreated CHB patients and to estimate their eligibility for antiviral therapy, according to the recommendations of the CPTG-CHB^7^. For this purpose, we collected data from Minas Gerais to determine which disease stage predominated in CHB patients assisted by the public health system and how many of them had indications for immediate treatment.

## METHODS

### Study Population and design

This descriptive cross-sectional study used secondary data (epidemiological and clinical-laboratory information, including sex, age, cities, and results of biochemical, serological, molecular, and histochemical tests) from the Central Public Health Laboratory (Fundação Ezequiel Dias - FUNED), collected from May 2012 to September 2013. Inclusion criteria: CHB patients, regardless of sex or age, residing in any city in Minas Gerais, under SUS care. Exclusion criteria: those who had already received CHB treatment and those lacking sufficient data. The sample size (n=548) was calculated assuming an expected proportion of 5% chronic carriers in an estimated 2,600,000 HBV population in Brazil (Seroprevalence of hepatitis B and C: a survey in Minas Gerais - 2009, unpublished data) and using a 95% confidence interval with a 2% error margin and a design effect of 1.0, plus a 20% loss rate.

**FIGURE 1:**
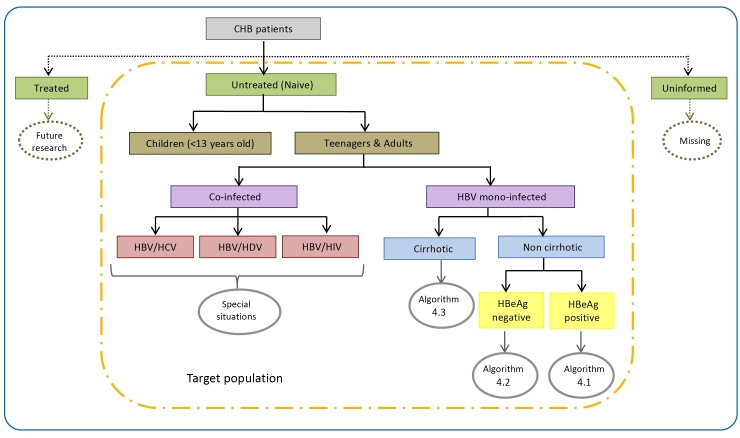
Allocation of CHB patients from Minas Gerais (Brazil) according to the clinical situations described in the “Clinical Protocol and Therapeutic Guidelines for the Treatment of Chronic Viral Hepatitis B and Co-infections” (2011).

Patient information was initially analyzed to identify and select those treatment-naive (target population of the study). Untreated patients were grouped according to their clinical and laboratory data for correct allocation in the respective algorithms (4.1, 4.2, 4.3) or segregated into special groups according to the CPTG-CHB (current version of data collection), as shown in Figure 1 ^7^. Following this, the candidates’ eligibility for antiviral therapy was analyzed (Table 1). The alanine aminotransferase (ALT) enzyme level was considered high if it was greater than the reference normal limit. The METAVIR liver biopsy scale is a combination of letters and numbers, where “A” corresponds to necroinflammatory activity (A0 = no activity, A1 = mild activity, A2 = moderate and A3 = severe) and “F” indicates the degree of fibrosis or cirrhosis (F0 = no fibrosis, F1 = portal fibrosis without septum, F2 = portal with rare septa, F3 = numerous septa without cirrhosis, and F4 = cirrhosis)^11^.

**TABLE 1:** Eligibility criteria for antiviral therapy indications according to the “Clinical Protocol and Therapeutic Guidelines for the Treatment of Chronic Viral Hepatitis B and Chronic Viral Coinfections” (version 2011, Brazil).

ELIGIBILITY CRITERIA FOR ANTIVIRAL THERAPY INDICATIONS IN BRAZIL (CPTG-CHB, 2011)		
Non-cirrhotic patients	Positive HBeAg	Altered ALT, regardless of other criteria. Biopsies showing inflammatory activity and fibrosis <A2 and / or <F2, regardless of levels of ALT.
	Negative HBeAg	Normal ALT, HBV-DNA ≥2,000 IU/mL, regardless of liver biopsy results.
		Altered ALT, HBV-DNA ≥200 IU mL and <2,000 IU/mL or biopsy showing inflammatory activity and / or fibrosis ≥A2 and / or ≥F2; or HBV-DNA ≥2,000 IU/mL, regardless of liver biopsy results.
Cirrhotic patients	Child-Pugh B e C	Any HBeAg condition and any levels of aminotransferase or HBV-DNA.
	Positive HBeAg	Any levels of aminotransferase or HBV-DNA and any Child-Pugh classification.
	Negative HBeAg	Child-Pugh A cirrhosis, altered levels of aminotransferase and / or HBV-DNA ≥200 IU/mL.
Children	Altered ALT	Any histological studies and provided other causes of hepatocellular aggression are ruled out.
	Positive HBeAg	HBV-DNA ≥20,000 UI/mL.
	Negative HBeAg	HBV-DNA ≥2,000 UI/mL.
HBV-HCV co-infection	Positive HBeAg	Any levels of aminotransferase or HBV-DNA
	Negative HBeAg	HBV-DNA ≥2,000 UI/mL.
HBV-HIV co-infection	Cirrose	Any levels of aminotransferase or HBV-DNA
	Positive HBeAg	HBV-DNA ≥2,000 UI/mL and / or altered ALT.
	Negative HBeAg	Histological changes with fibrosis ≥F1 to F4 or patients without fibrosis, but with necroinflammatory activity ≥A2.

Legend: ALT: alanine aminotransferase; HBeAg: hepatitis B antigen “e” virus; HBV-DNA: hepatitis B virus-deoxyribonucleic acid; HCV: hepatitis C virus; HIV: human immunodeficiency virus; CPTG-CHB: Clinical Protocol and Therapeutic Guidelines for the treatment of Chronic Viral Hepatitis B and Co-infections.

### Ethical considerations

This study was part of the “Laboratory surveillance applied to disease control and management of patients infected with HBV, HCV, and/or HIV among SUS users in Minas Gerais” project, which was approved by the Research Ethics Committee (approval number: 4.434.487) and conducted in accordance with the National Health Council of Brazil (Resolution 466/12) and the Helsinki Declaration (1964).

### Statistical analysis

Analyses were performed using Epi Info^TM^ software, v.7.2.1.0 (CDC, Georgia, USA), with results expressed as median and interquartile range (IQR 25% -75%), absolute frequencies, and proportions (%). The eligibility rate estimate for the antiviral therapy indications was calculated by dividing the absolute number of untreated CHB patients (who met the criteria defined in CPTG-CHB) by the total number of untreated CHB patients. The QGIS software v.3.10 (QGIS Development Team) was used to represent the spatial distribution of untreated CHB patients. The Strengthening the Reporting of Observational studies in Epidemiology **(**STROBE) guidelines were used in this study^12^.

## RESULTS

**FIGURE 2:**
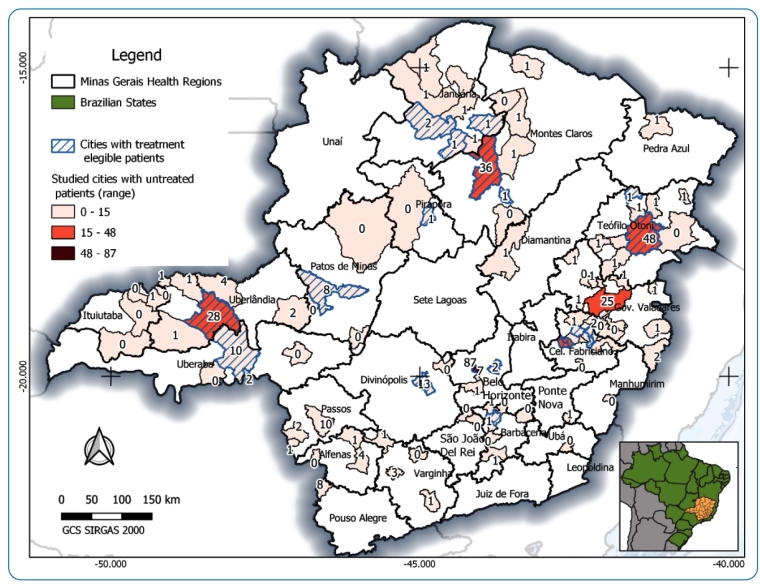
Geospatial location of untreated CHB patients by health region in the Minas Gerais state (Brazil), according to the eligibility criteria defined in the “Clinical Protocol and Therapeutic Guidelines for the Treatment of Chronic Viral Hepatitis B and Co-infections,” from May 2012 to September 2013.

Altogether, 670 HBV patient records assisted by 63 SUS physicians were included from 101 (11.8%) municipalities in Minas Gerais, Brazil (Figure 2). More than half (372; 55.5%) of the CHB patients were men, with an average age of 41 years (IIQ: 32-50) and an age range of 0-84 years. Of the 461 included patients (68.8%) in the study who had not yet received HBV treatment, only 377 were evaluated for antiviral therapy indications, although periodic monitoring of the disease (HBV-DNA and ALT) was performed in only three of these cases. On the other hand, 180 (26.9%) patients who had already undergone antiviral therapy and the remaining 29 (4.3%) who did not have information on their eligibility for antiviral therapy; thus, they were excluded from the analysis.

Overall, the untreated CHB patients (candidates for antiviral therapy in Brazil) were mainly female (246; 53.4%), non-pregnant (239; 97.2%), and adults (median age: 40 years; IQR: 31-49). The predominant clinical-laboratory picture was 426 (98.2%) monoinfected HBV patients, of which 396 (92.3%) had negative HBeAg serology, 385 (91.7%) had positive anti-HBe, and 333 (93.8%) had a negative anti-HBc IgM marker. From the molecular point of view, 145 (46.9%) of these cases had not yet received HBV quantification, another 12 (3.9%) had undetectable HBV-DNA levels and the remaining 152 (49.2%) had low viral loads (median 2.71 log IU/mL and IQR: 1.73-3.45). The ALT levels were normal in 359 patients (81.2%). A clinical diagnosis of cirrhosis was described in 15 patients (3.4%). Liver biopsy results were available for 20 of 440 patients with available data (4.6%), with no histological evidence of necroinflammatory activity <A2 in 9 (81.8%) and fibrosis <F2 in 9 (69.2%). Child-Pugh scores were not reported. The data showed that only three (0.7%) patients had records of regular monitoring of liver disease, both by HBV-DNA quantification and biochemical ALT measurement performed at intervals of six months (in two patients) or annually (in one).

**FIGURE 3:**
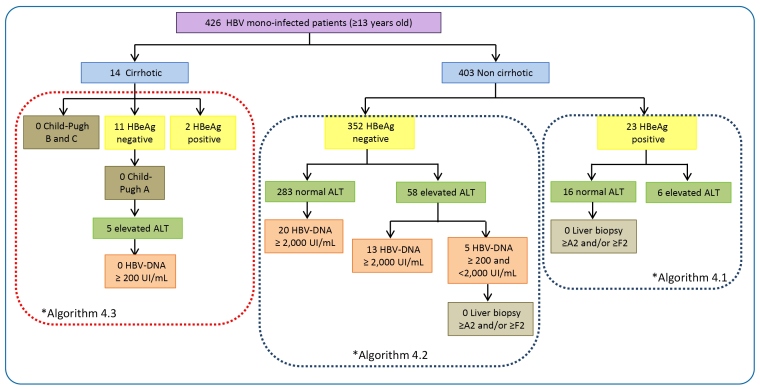
Distribution of untreated CHB patients that were over 13 years of age, including mono-infected and non-cirrhotic candidates for antiviral therapy, according to the different “Clinical Protocol and Therapeutic Guidelines for the Treatment of Chronic Viral Hepatitis B and Co-infections” algorithms (version 2011). Patients were evaluated from May 2012 to September 2013 at the Central Laboratory of Public Health of Minas Gerais (Brazil). *The number of individuals differs from the total due to lack of data.

**FIGURE 4:**
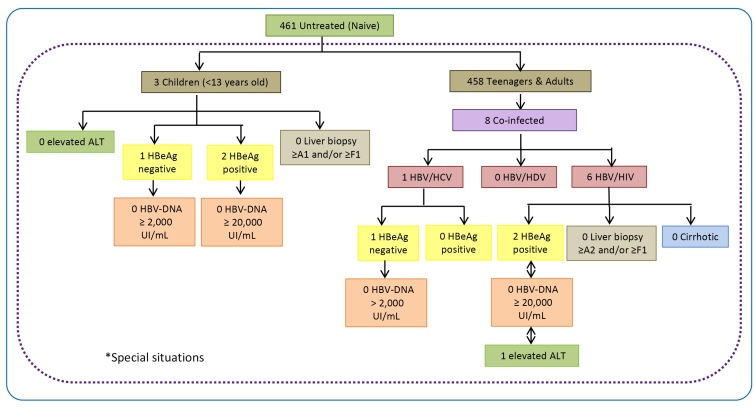
Distribution of untreated CHB patients that were classified as ‘special situation’ candidates for antiviral therapy according to the “Clinical Protocol and Therapeutic Guidelines for the treatment of Chronic Viral Hepatitis B and Co-infections” algorithms (version 2011). Patients were evaluated from May 2012 to September 2013 at the Central Laboratory of Public Health of Minas Gerais (Brazil). *The number of individuals differs from the total due to lack of data.

Considering the target population of 461 untreated CHB patients that were initially candidates for antiviral therapy, 43 (9.3%) met the criteria for treatment as defined by the CPTG-CHB (Figures 3 and 4). The findings showed that most of those eligible to start treatment were men (25; 58.1%) over 18 years old, with a median of 36 years (IQR: 28-44). The main clinical and laboratory findings of the eligible patients were HBV monoinfection and no cirrhosis (41; 95.4%). They had negative HBeAg (396, 92.3%), and seroconversion to positive anti-HBe was observed in 385 patients (91.7%). Viral load tests were performed on 36 (83.7%) eligible patients: one had an undetectable HBV-DNA level, and the others had moderate HBV-DNA concentrations (median 3.89 log UI/mL and IQR: 3.56-4.45). In addition, serum ALT levels were elevated above the upper limit of normal in 21 patients (48.8%). Among the eligible patients, liver biopsy was performed in 7 of 43 (17.1%) patients, with no significant histological evidence of necroinflammatory activity present in 6 (2 A0 patients and 4 A1 patients), and presence of fibrosis in 5 (4 F0 cases and 1 F4 case). There were no reports of Child-Pugh scores in any of these patients.

## DISCUSSION

More than 15 years has passed since the national implementation of the CPTG as a public health tool for CHB management in Brazil, and yet there are no detailed studies assessing whether the predictors for the timely initiation of antiviral treatment are being effectively applied in routine clinical practice^9^. This is the first study to address the main clinical and laboratory characteristics of CHB patients that are naïve to treatment and attended by the SUS in Minas Gerais (Brazil). For this purpose, the actual adherence to the pretreatment recommendations described in the CPTG-CHB was evaluated using the results from the laboratory tests necessary to determine the stage of the disease and indicate the beginning of therapy. As a secondary outcome, the proportion of CHB patients eligible for antiviral treatment was estimated according to the criteria established in the current guidelines^7^. It is believed that such information can assist in the development of hypotheses regarding the patterns of occurrence of this infection type, as well as indirectly support the planning and improvement of actions and services related to ideal CHB management in the public sphere.

This study highlights the high adherence rate of SUS physicians to the CPTG recommendations in regards to the initial practical conduct of screening and diagnosing CHB patients, which is applied on a daily basis. The collected data indicated that approximately 90% of the basic laboratory tests established to characterize the disease stage were adopted. In contrast, more advanced tests, such as molecular diagnosis for viral quantification (HBV-DNA), have only been performed in approximately one-third of these patients. In addition, among those in whom an examination would be optional to clarify their diagnosis, few patients underwent liver biopsy (10.0%), and in approximately 50% of the biopsies performed, liver histology was not available. It is noteworthy that since 2002, the entire clinical and diagnostic methodology established by the CPTG-CHB has been offered by the SUS without any additional costs to patients^7^. Knowledge of the current disease stage (based on clinical, biochemical, and virological tests) is a fundamental prerequisite for assertive therapeutic decision-making^5^ ^-^ ^7^ ^,^ ^13^. Therefore, the lack of information regarding clinical and laboratory results leads to a certain methodological limitation, likely leading to an underestimation of the real proportion of untreated patients who could be eligible for HBV therapy according to the applied guidelines.

Not all untreated CHB patients require immediate treatment, and continued periodic monitoring is crucial to identify those who would benefit from antiviral therapy in the future. Given the various clinical contexts throughout their lives, some of these patients may experience changes in the stage of their disease and in their eligibility criteria for treatment. Thus, ideal CHB management requires regular follow-up of patients, even when they are asymptomatic, in order to determine the possible progression of liver disease and the opportune moment for the introduction of specific therapies^8^. Thus, laboratory checks, particularly HBV-DNA and ALT, serve as parameters for detecting any indicative fluctuations of disease worsening in patients initially not eligible for treatment, as expressed by several other available guidelines^5^ ^-^ ^7^ ^,^ ^13^ ^,^ ^14^. The data analyzed in this study, regardless of the algorithm or special situations described in the CPTG-CHB, suggest that practically none of the patients (0.65%) received regular laboratory monitoring. These findings reflect practical conducts that are outside of the recommendations for CHB management within the integrative care services in the SUS.

However, these findings have recurred in several recent studies addressing this topic. These results are similar to those reported by Juday *et al*., where less than one-third of untreated CHB patients received adequate laboratory follow-ups in the USA, suggesting that antiviral therapy initiation could also have been delayed. This leaves patients at risk of complications resulting from the disease, such as cirrhosis and hepatocellular carcinoma^15^. Low adherence to CHB guidelines in clinical practice has been observed in studies among American, Spanish, and Chinese physicians, in which less than half of the professionals were reported to have complied with the recommendations, particularly with the requirement for regular disease monitoring, with high variability according to the specific test employed^16^ ^-^ ^18^. More frequent monitoring can increase HBV treatment indications, avoiding delays in starting treatment and, therefore, reducing the incidence of complications associated with this disease^15^ ^,^ ^16^.

The results showed that the majority of patients had not yet been treated for HBV, approximately 90% had a negative HBeAg serological pattern, and less than 4% had a diagnosis of cirrhosis, with a slight predominance of mono-infected women (53.4%) with a median age of 40 years. This profile corroborates with other Brazilian studies, although the CHB prevalence in relation to the sex of patients may differ in some regions of the country. Pacheco and colleagues evaluated 527 patients in two reference hepatitis outpatient clinics and reported that 86.1% of patients were HBeAg-negative, female (57.0%), with an average age of 40 years in the North region (Acre). This is in contrast to the data from the Northeast region (Bahia), where there was a predominance of men (59.3%) with an average age of 44 years^10^. The data found were also different from studies carried out in the Southeast region, in which high proportions of negative HBeAg serology (70%) were reported; this was more frequently (64%) seen in men with an average age of 42 years in São Paulo^19^. In patients at the Hepatitis B Outpatient Clinic in Ribeirão Preto, more than 60% of HBeAg-negative patients were male, and the average age was 38 years^20^. These variations can have several explanations, including the time of diagnosis, the time of infection, the different regional rates of HBV prevalence, and the various forms of viral transmission.

These findings were also similar to those of previous studies reporting a predominance of negative HBeAg markers in older HBV-infected patients. This serological pattern typically represents a late course of CHB and, in recent decades, represents the majority of cases in some Western European countries such as France, Portugal, Germany, Italy, and Spain, as well as in Greece^21^ ^-^ ^26^. However, the clinical picture differs from that of patients recruited for clinical trials of antiviral therapy, which does not always represent the types of patients commonly found in clinical practice. CHB is defined by this serological pattern and characterized by persistently normal levels of transaminases, low concentrations of HBV-DNA (usually <2,000 IU/mL), and low histological liver damage. This has been shown to be a trend in other countries in Europe, Asia, and the United States, mainly due to the aging of the infected population^22^. These patients are considered inactive CHB carriers and generally have a more favorable prognosis, with less hepatic histological damage and less frequent progression of the disease, particularly in women, which can persist for years or even a lifetime. However, in approximately 20% to 30% of inactive carriers, this pattern may change to reactivation of the disease (ALT elevation and high levels of HBV-DNA, moderate histological activity, occasionally promoting positive anti-HBe); thus, periodic monitoring of all CHB patients is essential^18^.

This study described the CHB treatment eligibility estimate among untreated patients who sought care in the public health system. Only 9.3% of the patients met the CPTG-CHB criteria established to initiate antiviral therapy. This eligibility proportion was similar to two other studies: one population-based study conducted in the Gambia, which reported that the eligibility rate for immediate antiviral therapy introduction was 4.4% among HBV individuals and 9.7% among blood donors, and another in West Africa, where the estimate was 10% among vulnerable positive HBsAg populations^27^ ^,^ ^28^. However, the rate obtained in the present study was lower than that reported by Spradling *et al*., in which 60% of CHB patients in public health settings in the USA were considered eligible^29^. This discrepancy can be explained by the selection of participants, in which one-third of the American cohort had active disease forms during the follow-up, in contrast to this study, where inactive patients predominated. It is estimated that approximately 25%-50% of CHB patients in an advanced stage of the disease are treatment-eligible under the applied guidelines^30^. These findings emphasize the importance of disease characterization using complete diagnostic techniques.

This study had some limitations. First, as this was a retrospective study, the loss of information (even in a low percentage) in certain laboratory tests may have underestimated the proportion of patients that were potentially eligible for antiviral therapy. Second, these results may not be applicable to the entire CHB population, as patients with private or commercial health plans are not represented in this dataset. However, the findings presented provide an insight into the provision of CHB patient care in the public health system, in which the selection bias can be considered limited since all residents in Brazil have universal and full access to the SUS service network. Another limitation is related to data collection, as if was performed in regions with a low HBV prevalence. Thus, these findings cannot be extended to other states or countries with higher prevalence rates, since this infection course can vary between geographic regions and according to the prevalent transmission route.

On the other hand, one of the strengths is the representative target population inclusion of CHB patients, which was spread over a considerable number of cities in Minas Gerais and covered different clinical conditions assisted by many physicians at all levels of health care within the SUS. In addition, the present study expanded on the current knowledge of CHB patient profiles in Brazil, determined the rates of execution of the recommended laboratory tests performed to characterize the predominant stage of the infection, and described the proportion of patients eligible for antiviral treatment.

In summary, most of the CHB patients who sought assistance in the Brazilian public health system were in the ‘inactive carriers’ stage, with a low proportion of initially eligible candidates for antiviral therapy. In addition, this study revealed inadequate adherence to the pretreatment clinical conduct that should be applied for patients not yet exposed to HBV treatment. It is worth emphasizing that according to the CPTG-CHB recommendations, laboratory monitoring regularity was almost absent, and it was highly variable for each specific test. Additional studies are needed to assess the barriers related to adherence to these guidelines, and the rates of treatment should be assessed through long-term CHB patient follow-ups.
